# Elevated temperatures and longer durations improve the efficacy of oxaliplatin- and mitomycin C-based hyperthermic intraperitoneal chemotherapy in a confirmed rat model for peritoneal metastasis of colorectal cancer origin

**DOI:** 10.3389/fonc.2023.1122755

**Published:** 2023-03-16

**Authors:** Roxan F. C. P. A. Helderman, Bella Bokan, Gregor G. W. van Bochove, Hans M. Rodermond, Elsy Thijssen, Wouter Marchal, Arezo Torang, Daan R. Löke, Nicolaas A. P. Franken, H. Petra Kok, Pieter J. Tanis, Johannes Crezee, Arlene L. Oei

**Affiliations:** ^1^ Department of Radiation Oncology, Amsterdam University Medical Centers (UMC) Location University of Amsterdam, Amsterdam, Netherlands; ^2^ Center for Experimental and Molecular Medicine (CEMM), Laboratory for Experimental Oncology and Radiobiology (LEXOR), Amsterdam, Netherlands; ^3^ Cancer Biology and Immunology, Cancer Center Amsterdam, Amsterdam, Netherlands; ^4^ Institute for Materials Research, Analytical and Circular Chemistry, Hasselt University, Diepenbeek, Belgium; ^5^ Oncode Institute, Amsterdam, Netherlands; ^6^ Department of Surgery, Amsterdam University Medical Centers (UMC) Location University of Amsterdam, Amsterdam, Netherlands; ^7^ Department of Surgical Oncology and Gastrointestinal Surgery, Erasmus MC Cancer Institute, Rotterdam, Netherlands

**Keywords:** colorectal cancer, peritoneal metastasis, hyperthermic intraperitoneal chemotherapy (hipec), orthotopic rat model, hyperthermia, oxaliplatin, mitomycin-C

## Abstract

**Introduction:**

In patients with limited peritoneal metastasis (PM) originating from colorectal cancer, cytoreductive surgery (CRS) followed by hyperthermic intraperitoneal chemotherapy (HIPEC) is a potentially curative treatment option. This combined treatment modality using HIPEC with mitomycin C (MMC) for 90 minutes proved to be superior to systemic chemotherapy alone, but no benefit of adding HIPEC to CRS alone was shown using oxaliplatin-based HIPEC during 30 minutes. We investigated the impact of treatment temperature and duration as relevant HIPEC parameters for these two chemotherapeutic agents in representative preclinical models. The temperature- and duration- dependent efficacy for both oxaliplatin and MMC was evaluated in an *in vitro* setting and in a representative animal model.

**Methods:**

In 130 WAG/Rij rats, PM were established through i.p. injections of rat CC-531 colon carcinoma cells with a signature similar to the dominant treatment-resistant CMS4 type human colorectal PM. Tumor growth was monitored twice per week using ultrasound, and HIPEC was applied when most tumors were 4-6 mm. A semi-open four-inflow HIPEC setup was used to circulate oxaliplatin or MMC through the peritoneum for 30, 60 or 90 minutes with inflow temperatures of 38°C or 42°C to achieve temperatures in the peritoneum of 37°C or 41°C. Tumors, healthy tissue and blood were collected directly or 48 hours after treatment to assess the platinum uptake, level of apoptosis and proliferation and to determine the healthy tissue toxicity.

**Results:**

*In vitro* results show a temperature- and duration- dependent efficacy for both oxaliplatin and MMC in both CC-531 cells and organoids. Temperature distribution throughout the peritoneum of the rats was stable with normothermic and hyperthermic average temperatures in the peritoneum ranging from 36.95-37.63°C and 40.51-41.37°C, respectively. Treatments resulted in minimal body weight decrease (<10%) and only 7/130 rats did not reach the endpoint of 48 hours after treatment.

**Conclusions:**

Both elevated temperatures and longer treatment duration resulted in a higher platinum uptake, significantly increased apoptosis and lower proliferation in PM tumor lesions, without enhanced normal tissue toxicity. Our results demonstrated that oxaliplatin- and MMC-based HIPEC procedures are both temperature- and duration-dependent in an *in vivo* tumor model.

## 1 Introduction

Hyperthermic intraperitoneal chemotherapy (HIPEC) is a cancer treatment that involves circulation of heated chemotherapeutic solution through the peritoneal cavity of patients with peritoneal metastasis (PM). These metastases can originate from several gastrointestinal or gynecological malignancies and have a poor prognosis ranging from 4-24 months (1–5).

Preceding HIPEC treatment, all visible macroscopic tumor lesions are removed during cytoreductive surgery (CRS). The aim of the HIPEC procedure is to eliminate residual (microscopically) tumor lesions that are left behind after CRS, as well as free floating tumor cells in the abdominal cavity. The combination of CRS with HIPEC is the only treatment option with curative intent for patients with PM. Several clinical studies showed a prolonged survival when CRS and/or systemic chemotherapy-based treatment was combined with HIPEC in patients with ovarian cancer (6), gastric cancer (7, 8), colorectal cancer (CRC) (9–11), malignant peritoneal mesothelioma (MPM) (12) or pseudomyxoma peritonei (PMP) (13).

In CRC patients with PM (PMCRC), a landmark trial randomizing between only fluoropyrimidine systemic chemotherapy or CRS followed by a mitomycin-C (MMC)-based HIPEC treatment (90 minutes at 41-42°C) and systemic 5-FU revealed a significant improvement in median overall survival of 12.6 *vs*. 22.2 months in the HIPEC arm (9). This benefit was confirmed in a comparative cohort study reported by Franko et al. using a similar HIPEC schedule (medium overall survival of 16.8 *vs*. 34.7 months) (10). Elias et al. introduced an oxaliplatin-based HIPEC treatment with shorter duration and reported much higher overall survival rates if compared to modern systemic chemotherapy alone in a non-randomized comparison (23.9 *vs*. 62.7 months) (11). These results show a clear beneficial effect of HIPEC compared to systemic treatment only. In some approaches intraperitoneal chemotherapy is repeated more than once, using MMC and oxaliplatin for patients with unresectable recurrent rectal cancer (14).

The efficacy of HIPEC largely depends on the treatment protocol based on no less than eight relevant HIPEC treatment parameters; type of drug, concentration of the drug, carrier solution, volume, temperature, duration, delivery technique and patient selection (15, 16). Unfortunately, a standardized HIPEC treatment protocol is lacking resulting in a large institutional variation in the application of HIPEC worldwide. In order to determine the effectiveness of HIPEC in the clinic, it is necessary to quantify the effect of each parameter separately in order to define a standardized HIPEC treatment protocol.

In response to negative HIPEC trials with 30 minute exposure to oxaliplatin, the effect of duration and temperature should be investigated during HIPEC procedures using both oxaliplatin or MMC for durations up to 90 minutes as often used in HIPEC (17–19). Preclinical research in a representative model is a powerful strategy to quantify these effects (20). The duration- and temperature-dependent effectiveness of several drugs were thoroughly explored in several cancer types in an *in vitro* setting, showing increased efficacy, suggesting that they are suitable for HIPEC (21–24). Although experiments performed on cells and organoids have advantages, they do not provide insights in the impact of HIPEC on the tumor microenvironment and on normal tissue toxicity, which makes the translation to the clinical setting difficult. A more realistic setting can be obtained by using a suitable animal model combined with an optimized HIPEC setup to guarantee well-controlled treatment settings for the parameter under study. A suitable animal model requires a representative tumor model which should be confirmed to contain metastases before conducting the HIPEC treatment and these metastases should be large enough to perform an adequate HIPEC procedure.

**Figure 1:**
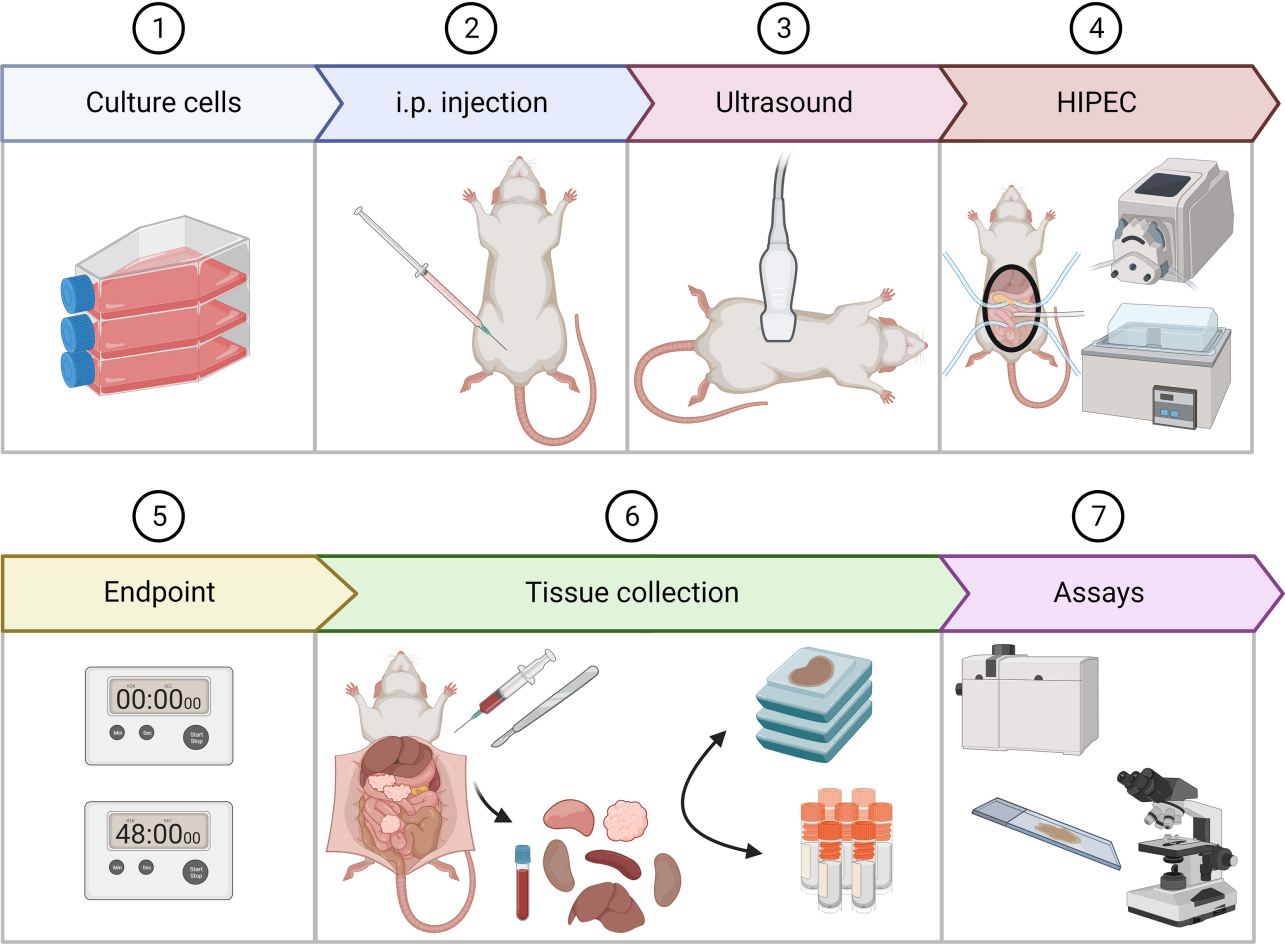
An overview of the experimental design. After rats were injected with 2x 10^6^ CC-531 cells, tumor outgrowth was followed using ultrasound. When tumors reached a size of 4-6 mm, the HIPEC procedure was performed. After treatment, rats were sacrificed 0 or 48 hours after treatment. Upon sacrifice, tissue collection was performed. Tumor lesions from 3 different regions, blood and healthy organs were collected in 4% formalin and embedded in paraffin or fresh frozen and stored at -80°C. Platinum uptake was measured using inductively coupled plasma mass spectrometry. Apoptosis and proliferation were assessed using immunohistochemistry. This figure is created with BioRender.com.

The aim of this study is to evaluate the direct effect of temperature and duration during oxaliplatin- and MMC-based HIPEC procedures in CRC cells and organoids and eventually in a PM confirmed rat model. An overview of the experimental setup is presented in **Figure 1**. WAG/Rij rats are injected intraperitoneal with CC-531 cells and ultrasound is applied to confirm tumor outgrowth in the peritoneum before the HIPEC treatment is conducted (25). HIPEC procedures are applied using oxaliplatin or MMC with a treatment duration of 30, 60 and 90 minutes with inflow temperatures at 38°C or 42°C. Samples are collected either immediately or 48 hours after treatment to study the effect on platinum uptake, apoptosis, proliferation and healthy tissue toxicity. These results should give insights towards an optimized HIPEC treatment protocol for CRC patients with metastasis in the peritoneum.

## 2 Materials and methods

### 2.1 Cell culture

The CC-531 cell line are colon carcinoma cells derived from WAG/Rij rats with a signature similar to the dominant treatment-resistant CMS4 type human colorectal PM. These cells were kindly provided by Prof. Dr. Ignace de Hingh, Catharina Cancer Institute (Eindhoven) and Roger Lomme, Radboud University (Nijmegen). This cell line was cultured in RPMI1640 medium (Gibco) containing 25 mM HEPES and supplemented with 10% fetal bovine serum (Gibco) and 1% penicillin/streptomycin/glutamine (Gibco). Cells were maintained at 37°C in a humidified atmosphere of 5% CO2 in air.

### 2.2 *In vitro* HIPEC-treatment

CC-531 cells were exposed to various concentrations of two chemotherapeutic agents under normal (37°C) and hyperthermic (42°C) conditions for 30, 60 minutes or 90 minutes (plus an additional 10-minute pre-heating). Stock-solutions of agents: oxaliplatin (Cayman Chemical Company) and MMC (Bio-connect) were stored at 4°C. Appropriate dilutions were freshly prepared for each experiment. Hyperthermia was performed by submerging the plates in a thermostatically regulated water bath supplemented with 5% CO2. After treatment, medium was replaced with fresh medium (without chemotherapy) and cells were incubated at 37°C in an atmosphere of 5% CO2 in air for the desired time of the specific assay.

### 2.3 Cell viability assay

Cell viability was assessed by PrestoBlue^®^ Cell Viability assays (Invitrogen). Cells were harvested and counted using an automated cell counter (Luna) and 20.000 cells were plated in each well of a 48-wells plate (Greiner). Plates were incubated overnight at 37°C and subjected to the *in vitro* HIPEC-treatment. After 2 days of incubation, the medium was replaced by 10 times diluted PrestoBlue^®^ Cell Viability Reagent in complete medium and incubated for 4 hours at 37°C. The extinction of every well was measured using a plate reader (Biotek synergy HTX) at 570 nm. Cell viability fractions were calculated by correcting the extinction of treated cells by the extinction of control cells.

### 2.4 Clonogenic survival assay

To assess long-term cell survival of treated and untreated cells, clonogenic survival assays were performed as described before (26). Cells were harvested and counted using an automated cell counter (Luna) and plated in appropriate density in 6-well plates (Greiner). Plates were incubated overnight at 37°C and treated as described above. After treatment the cells were incubated for 10 days for colony formation. Colonies were fixed by 6% glutaraldehyde and stained with 0.05% crystal violet. When a cell has the clonogenic capacity to dived into a cluster of 50 cells or more, it was score as a colony. Surviving fractions were calculated by correcting the plating efficiency of untreated control cells.

### 2.5 Gene expression profiling

RNA was extracted from CC-531 cells, CC-531 organoids and CC-531 ex vivo tumor tissue grown in the peritoneum of WAG/Rij rats using the RNA isolation kit (Bioke). Three different passages per sample type were used resulting in a total of 9 samples. RNA concentrations were measured using NanoDropTM 2000 instrument (Thermo Fisher Scientific) and RNA integrity was assessed using TapeStation (Agilent). Library preparation was constructed using KAPA mRNA Hyperprep following by RNA-seq performed on an Illumina HiSeq 4000.

To assess the quality of raw data, FastQC (v.0.11.9) (27) and MultiQC (v.1.9) (28) were used prior to processing the data. The first 11 bases were trimmed from each read and low-quality bases (Phred score < 20) were removed using Cutadapt (v1.18) (29). To align the sequences STAR (v.2.7.4.a) was applied. The reference genome used in this step was mRatBN7.2. Finally, the number of counts per gene was obtained by STAR.

### 2.6 Consensus molecular subgroups classification

Publicly available datasets of established cell lines of human colorectal cancers were collected (GSE36133, GSE100478, GSE59857) and combined using the genes profiled in all three datasets. To eliminate the batch effects, datasets were normalized employing normalize.quantiles function of preprocessCore package (30) and comBat function (sva R package) (31). These samples were CMS stratified as previously described (32) using the classifier developed by Linnekamp et al. (33). To define signatures for each CMS subtype, 12 samples per each CMS were randomly selected from the combined set and differential expression analysis was performed using limma package in R (34). Genes with p value < 0.05 and positive fold changes were assigned to corresponding CMS subtype. Then the expression value of genes in these signatures were averaged per CMS.

Prior to CMS classification, counts per gene for all CC-531 samples were log2-transformed and quantile-normalized. Genes with average of log2 expression < 4 were removed from the analyses. The expression of each gene was next average across the samples of same treatment, to summarize data. Finally genes overlapping in this set and the CMS signatures were obtained and the top 100 genes per CMS ranked by p value were used to calculate the correlation between each group of samples and different CMS subtypes.

### 2.7 Immunohistochemistry on cells

To assess changes in proliferation or apoptosis in the cells after treatment, IHC stainings for Ki-67 and cleaved caspase-3 were performed. Cells were seeded on sterile cover slips (21x26 mm) placed in 60 mm cell culture dishes and were incubated overnight at 37°C. Cells were treated as described above. After treatment, medium was refreshed and cells were incubated for 48 hours. Cells were washed with PBS and fixed with 4% paraformaldehyde for 20 minutes. After washing the cells three times with PBS, cells were permeabilized with PBS containing 0.1% Triton X-100 and 1% fetal goat serum (TNBS) for 30 minutes. Slides were incubated with the primary antibody recombinant anti-Ki67 antibody 1:200 (anti-rabbit, Abcam) or cleaved caspase-3 antibody 1:100 (anti-rabbit, Cell Signaling) for 1 hour at room temperature. After washing the cells three times with PBS slides were incubated with secondary antibody goat-anti-mouse Cy3 (Jackson ImmunoResearch) diluted 1:100 in TNBS for 30 minutes in the dark. Cells were washed twice with PBS and nuclei were stained with DAPI (2.5 µg/ml) for 20 minutes. Coverslips embedded in vectashield were sealed on microscope slides. Digital image analysis was performed using Leica LAS-X software (Leica). Pictures of at least 150 cells from three independent experiments were obtained using Leica DM6 microscope quipped with a CCD camera.

### 2.8 Animals and housing

The effect of temperature and duration during HIPEC was examined in 130 7-9 week-old female WAG/Rij rats (Charles River Laboratories Research models and services). The rats were housed in individually ventilated cages (IVC-cages) (four per cage) with corn cob bedding material under standardized conditions: temperature 21°C, relative humidity 50–60%, 12 h light/12 h dark and with free access to standard water and food without additions. All animals were acclimatized for at least 1 week before the start of the experiment. All experiments were approved by the Dutch Central Committee of Animal Experiments, with approval code AVD1180020174184, received on 14 February 2019, and carried out in accordance with the Dutch Animal Welfare Act 1997.

### 2.9 Orthotopic PM rat model

Tumor lesions throughout the abdominal cavity were induced by intraperitoneal injections with CC-531 in 8-10 week-old WAG/Rij rats. CC-531 cells were cultured for at least two passages after being thawed in RPMI medium (Gibco) containing 25 mM HEPES, supplemented with 10% fetal bovine serum (FBS) (Gibco) and 1% penicillin/streptomycin/glutamine (Gibco). Cells were maintained at 37°C in a humidified atmosphere of 5% CO2 in air. The cell line was verified for authenticity and routinely tested for mycoplasma infection before the start of the experiment.

Cells were collected upon 60-70% confluency using trypsin (Gibco) and washed twice with PBS before concentrated to 2 × 10^6^ cells/mL. Per rat 1 mL of the cell suspension in culture medium containing 10% FBS was injected intraperitoneally in the right lower part of the abdominal cavity.

### 2.10 Ultrasound for PM confirmation in rats

After injection, ultrasound was applied weekly to assess the tumor outgrowth in the rats, starting 7 days after cell injection. Ultrasound was performed using the CX50 ultrasound (Philips) with probe L15-7io (Philips) on B mode in focal zone 1-2 with a frequency of 42 Hz and a gain of 90. Rats were anesthetized with 0.5-2.5% isoflurane in 100% oxygen using an isoflurane anesthesia vaporizer. The abdominal skin was shaved and cleaned with 70% ethanol before applying ultrasound gel (Aquasonic 100). A validated stepwise protocol was used to make sure that each region of the abdominal cavity was covered (25). It is important to check for remarkable changes and abnormalities in size, shape or location of organs during the procedure. On ultrasound, tumor lesions are mostly identified as superficial dark (hypoechoic) rounded structures, sometimes surrounded by a white (hyperechoic) halo. HIPEC was performed when most tumors reached a size of 4-6 mm.

### 2.11 HIPEC setup

**Figure 2:**
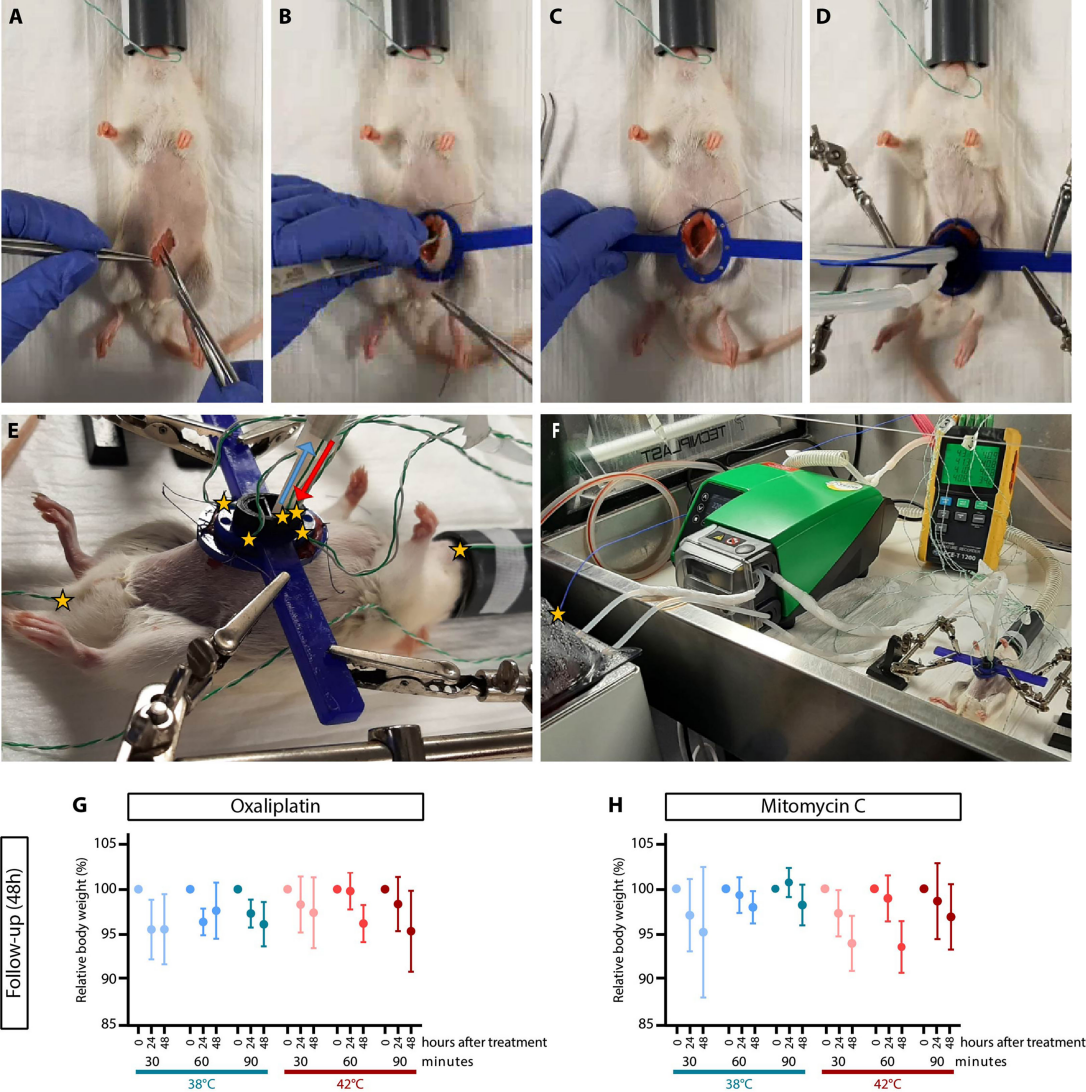
HIPEC treatments in rats did not affect body weight. To allow access to the peritoneum, a small incision was made **(A)** and the abdominal wall was attached to a plastic retractor ring with sutures **(B, C)**, enabling the placement the semi-open in/outflow HIPEC construction **(D)**. Temperatures probes were placed in the esophagus, inflow (red), outflow (blue), each of the four quadrants, rectum **(E)** and in the stock solution **(F)**. Temperature spots were indicated by yellow stars. The average body weight immediately after, 24 hours and 48 hours after HIPEC with oxaliplatin **(G)** and mitomycin-C **(H)** is shown for a non-heated treatment procedure (38°C) and a heated treatment procedure (42°C). Means ± SD of 4-5 animals were presented.

During the HIPEC procedure, a heated perfusate solution was circulated through the abdominal cavity using a closed circulation-circuit which was created by placing two pumpsil tubes through a roller pump (530 Un/R, Watson-Marlow). One end was placed in the perfusate solution placed in a thermostatically water bath (aqualine AL12, Lauda) and the other end was connected to our in-house semi-open in/outflow HIPEC construction (35). To avoid an unobstructed flow due to a clogged outflow catheter, an in/outflow construction was created to separate the outflow from the peritoneal cavity. During the entire procedure, the temperature was monitored at nine different spots (**Figure 2E**): in the perfusate stock solution, the in- and outflow catheters, in each of the four quadrants, in the esophagus and rectal by using a multi-channel data-logging digital thermometer (PCE-T 1200, PCE-instruments).

### 2.12 HIPEC procedure

To evaluate the direct effect of HIPEC on PM, CRS was not applied right before the HIPEC procedure to make certain small tumors were present in the abdominal cavity right after treatment to collect for analysis.

Before the start of the procedure, the perfusate solution was prepared and pre-heated using a thermostatically water bath. In order to achieve temperatures of 37°C or 41°C in the peritoneum, the inflow temperature were set on 38°C or 42°C. Rats were treated with 150 mg/m^2^ oxaliplatin (Cayman Chemical Company) of 35 mg/m^2^ MMC in 500 mL physioneal 40 glucose 1.36% (Baxter) carrier solution.

The procedure was performed in a biosafety cabinet as described before (35) **Figure 2F**. Rats were anesthetized with 0.5–2.5% isoflurane in 100% oxygen using an isoflurane anesthesia vaporizer and placed on a heating mat to avoid decreasing body temperatures. The abdominal skin was shaved and disinfected with betadine before a midline incision of approximately 4 cm was made **Figure 2A**. The abdominal wall was attached to a plastic retractor ring *via* 4 sutures **Figures 2B, C**. The in/outflow construction, containing four perforated inflow tubes and one outflow catheter, was placed through the retractor. The retractor and in/outflow construction was fixed with two holders (Third-hands, Toolcraft) to ensure proper positioning **Figure 2D**.

To start the HIPEC, the roller pump was set on 220 RPM, corresponding to 1.6 mL/s. After approximately 5-10 min the desired temperature was reached in all four quadrants. Subsequently, HIPEC was applied for 30, 60 or 90 minutes. During the procedure, the body temperature was kept stable using the heating mat when using an inflow temperature of 38°C. At an inflow temperature of 42°C, the heating mat was removed and partial cooling of the tail with a tissue soaked with 70% ethanol was applied when the temperature in the esophagus exceeded 38°C.

At the end of the HIPEC procedure, the inflow catheter was removed from the perfusate solution to remove remaining fluid from the abdomen. For each animal, 1 mL peritoneal fluid was collected. The in/outflow construction and retractor ring were removed. The abdominal wall and skin were closed with single sutures and disinfected with betadine in order to follow-up the animals and to collect tissue 48 hours after treatment. The other half of the animals were directly sacrificed in order to collect tissue right after surgery (0 hours).

### 2.13 Supportive care

To prevent inflammation and reduce postoperative pain, rats were treated with carprofen rimadyl (50 mg/mL, Patterson Veterinary). Two days before the HIPEC procedure, carprofen was supplied *via* the drinking water at a concentration of 0.0067 mg/mL, until two days after surgery. Right after the HIPEC procedure, only rats with a 48 hours follow-up received subcutaneously injection with a high dose of carprofen (5 mg/kg) to increase the systemic carprofen levels. Food was made accessible by placing food in the cage.

### 2.14 Treatment conditions, postoperative complications and survival

HIPEC was applied in 130 rats using oxaliplatin or MMC for 30, 60 or 90 minutes with an inflow temperature of 38°C or 42°C. Rats were sacrificed immediately after the HIPEC treatment (0h) to assess the direct effects of the HIPEC procedure or two days after treatment to assess the effects at a longer time point post-HIPEC (48h). The goal was to include 5 animals reaching the endpoint per group, which was completed for 22 of the 25 groups (**Table 1**). Animals with a two day follow-up were checked daily for signs of discomfort such as: weight loss (>10%), inactive behavior and an open fur. In total eight animals were sacrificed earlier after treatment because of signs of discomfort were noticed. In the oxaliplatin 30 min 38°C, 30 min 42°C and 90 min 42°C treatment groups, animals were sacrificed 24, 1 and 8 hours after treatment. Animals not reaching the endpoint were treated with MMC 60 min 38°C, 60 min 42°C, 90 min 42°C and were all sacrificed 24 hours after treatment.

**Table 1:** Treatment groups indicating the number of included rats/total number of treated rats.

Follow-up	0h						48h						Number of rats
**Inflow temperature**	38°C			42°C			38°C			42°C			
**Duration (min)**	30	60	90	30	60	90	30	60	90	30	60	90	
**Untreated**	5												5
**Oxaliplatin**	5	5	5	5	5	5	5	5	5	5	5	5	60
*Excluded*	–	–	–	–	–	–	1	–	–	1	–	2	4
**Mitomycin C**	5	5	5	5	5	5	5	4	4	5	4	5	57
*Excluded*	–	–	–	–	–	–	–	2	–	–	1	1	4
**Total number of rats**													130

### 2.15 Scoring extent of disease

**Figure 3:**
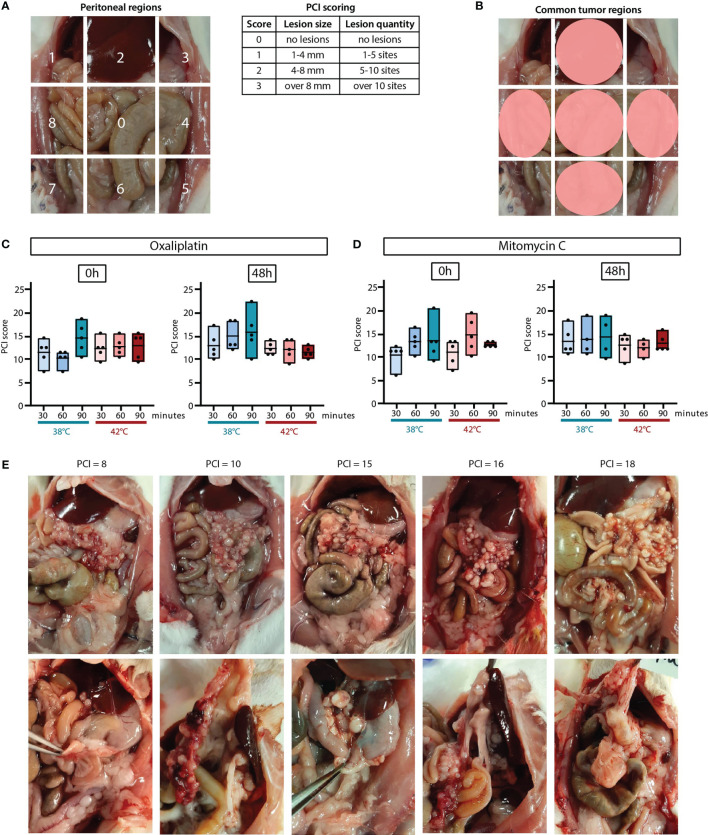
The extent of disease was scored upon sacrifice using the peritoneal carcinomatosis index (PCI) score. The number and size of the tumor lesion were scored in 9 regions with a score of 0, 1, 2 or 3 **(A)**. Tumor lesions were most common in regions 2, 0, 4, 6 and 8 **(B)**. PCI scores measured 0 hours and 48 hours after HIPEC treatment with oxaliplatin **(C)** or MMC **(D)**. All values of 4-5 animals are presented with the min/max ± means. Representative pictures with corresponding PCI scores of the abdomen with visible tumor lesions on the omentum (upper panel) and other regions (lower panel) **(E)**.

The extent of disease was measured using the peritoneal carcinomatosis index (PCI) upon sacrifice as described before (25). In short, the peritoneal cavity of the rats was divided in nine regions, and for each region, the size of the biggest nodule was scored as well as the number of lesions, with a score ranging between 0 – 3 (**Figure 3A**).

### 2.16 Tissue collection

Peritoneal fluid was collected right after each HIPEC procedure. Tumor nodules, organs and blood were collected upon sacrifice. Blood samples were centrifuged for 10 minutes at 1500 rpm at room temperature. The remaining plasma, peritoneal fluid and half of the collected tumor nodules and organs were fresh frozen and stored at -80°C until the day of inductively coupled plasma mass spectrometry analysis. The other half of the tumor nodules and organs were fixed in 4% formalin, dehydrated and embedded in paraffin for IHC stainings.

### 2.17 Inductively coupled plasma mass spectrometry

Platinum quantification was performed using a previously validated ICP-MS assay (36). Key parameters of the analytical methodology such as calibration curve linearity, detection limits and RSD values of QC standards were found to be in line with the values reported in the validated study. The analysis was carried out on a Perkin Elmer NexION 350S apparatus, equipped with the Syngistix software version 2.5 and an ESI Prep-Fast delivery system controlled by the ESI SC software version 2.9.0.202.

Tumor nodules were digested in 30% hydrogen peroxide (Merck KGaA, Darmstadt, Germany) and in 69-70% nitric acid (J.T. Baker, Avantor Performance Materials, Pennsylvania, United States), mixed in a 1:4 ratio by volume, using an Ethos Up microwave digestion system (Milestone). Digestion was performed according to the following schedule: 15 minutes, 1800 W, heating up to 200°C, followed by an isothermal period of 15 minutes at a constant temperature of 200°C and finally a cool down period. Afterwards the mixture was transferred quantitatively and adjusted to 50 mL using volumetric flasks, and 10-fold diluted before ICP-MS analysis using Milli-Q water (18 MΩ cm).

Sample preparation of the plasma and peritoneal fluid samples involved a simple 1/75 dilution in 0.5% nitric acid. Tb was added as an internal standard with a final concentration of 20 ng/mL Tb for each sample.

For the analysis, isotopes of Pt and the internal standard terbium (Tb) were monitored at m/z Pt 194, Pt 195 and Tb 159. The Pt 194 and 195 results were checked for consistency and subsequently the m/z Pt 194 data are used for quantification. The Tb internal standard recovery was systematically found between 86.4 and 104.9%. The Pt concentration in the tumor nodules was expressed as ng/mg wet tissue. Pt concentration in plasma and peritoneal fluid samples was expressed as μg/mL.

### 2.18 IHC on tumor and organ tissue

Proliferation and apoptosis in the tumor nodules and organs were detected with IHC stainings for Ki-67 and cleaved caspase-3, respectively. Sections of 4 µm were cut, deparaffinized and heat-induced antigen retrieval was performed with 1x Sodium Citrate pH 6 at 98°C, followed by peroxidase blocking. Slides were incubated with the primary antibody Recombinant Anti-Ki-67 antibody 1:200 (anti-rabbit, Abcam) or cleaved caspase-3 1:200 (anti-rabbit, Cell Signaling) overnight at room temperature. The next day, sections were incubated with the secondary antibody Poly-HRP-anti-Rabbit IgG (Immunologic) for 60 minutes at room temperature. Afterwards, the sections were stained with PowerDAB (Immunologic), counterstained with hematoxylin (Fluka) and mounted with Pertex. Representative pictures were taken with a light microscope (Olympus) at a magnification of 40x. Percentage positive staining was analyzed using the software program QuPath version 0.2.3.

### 2.19 Statistics

Statistical analyses were performed using R Studio software. The data were checked to for normal distribution. Comparison of independent treatment groups for cell viability, clonogenicity, organoid viability, platinum uptake, proliferation and apoptosis were calculated using two-way ANOVA, a parametric test. *Post hoc* analysis was performed using Tukey test. Significant p values are reported as * <0.05, **<0.01 and ***<0.001.

## 3 Results

### 3.1 The effect of oxaliplatin and MMC is temperature- and duration-dependent in rat CRC cells

**Figure 4:**
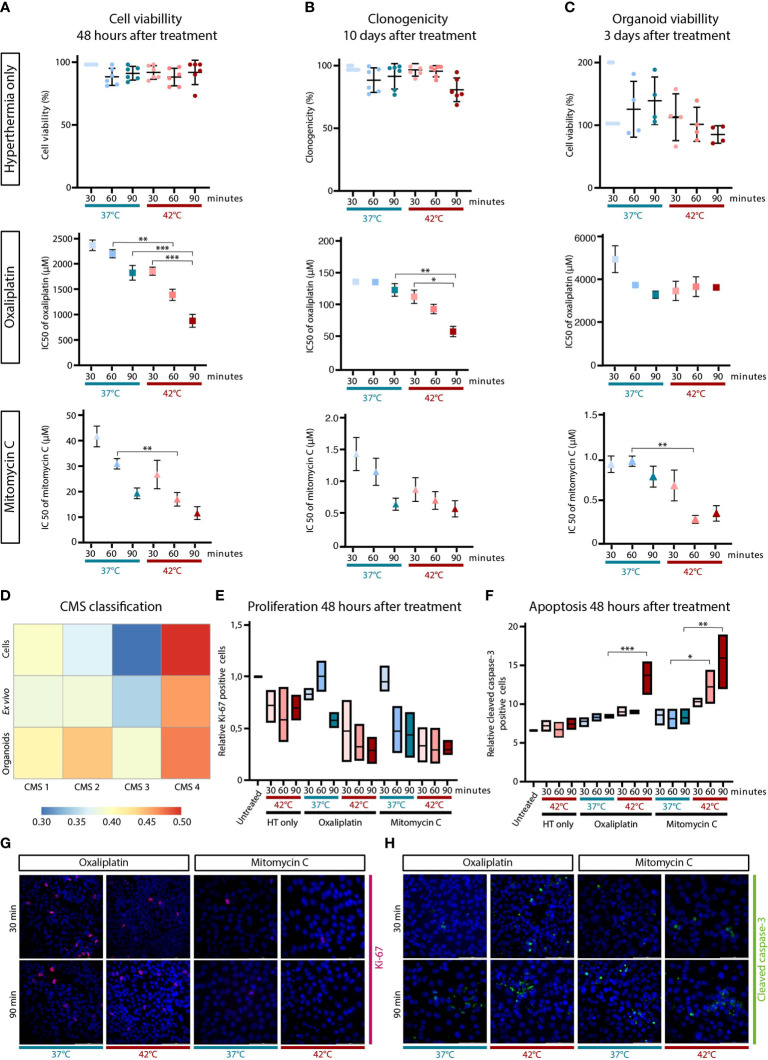
Hyperthermia reduces cell viability and clonogenicity, and increases apoptosis in oxaliplatin or mitomycin-C (MMC) treated CC-531 cells. CC-531 cells were exposed to oxaliplatin or MMC under normal (37°C) and hyperthermic (42°C) conditions for 30, 60 or 90 minutes. The effect of hyperthermia only, oxaliplatin and MMC was assessed using cell viability **(A)**, clonogenic **(B)** and organoid viability **(C)** assays performed 48 h, 10 days and 72 h after treatment, respectively. Data represent the means ± SD of 3 independent experiments. RNA sequencing was performed on the CC-531 cells to define the CMS subtype **(D)**. Proliferation **(E)** and apoptosis **(F)** levels were studied using immunohistochemistry, which was performed 48 h after treatment. Min/max ± means of 3 independent experiments are presented. Representative pictures are depicted with in pink Ki-67 **(G)** and in green cleaved caspase-3 **(H)** positive cells. Significant p-values are reported as * <0.05, **<0.01 and ***<0.001.

First, it is important to define the CMS subtype of the CC-531 cells, organoids and ex vivo tumors, since the CMS4 subtype was observed to be significantly enriched in PMCRC patients (37). RNA sequencing show that in all samples the correlation of models with CMS4 was the highest, confirming that the CC-531 samples have a CMS4-like subtype and therefore, results are relevant in the clinical setting (**Figure 4D**).

The effect of temperature and duration on the effectiveness of HIPEC was evaluated in CC-531 cells treated with a range of various concentrations oxaliplatin or MMC with or without hyperthermia with a duration of 30, 60 or 90 minutes. Hyperthermia only resulted in a minimal viability decrease 48 hours after treatment, but IC50 values of both oxaliplatin and MMC treatment decreased when treated at 42°C (**Figure 4A**). Also extending the treatment duration resulted in lower IC50 values. The long-term effect was assessed by performing clonogenic assay 10 days after treatment. Similar results were obtained with a minimal decrease after hyperthermia only, except for 90 minutes at 42°C after which only 84% of the cells survived (**Figure 4B**). For both drugs the IC50 values are decreasing when treated at a higher temperature and/or longer duration.

Subsequently to 2D cell cultures, CC-531 cells were cultured in matrigel in order to develop organoids and to evaluate the effect on 3D cell culture. The organoid viability 3 days after treatment are presented in **Figure 4C**. Results obtained for MMC in the CC-531 organoids are similar, but the oxaliplatin concentration needed to achieve the IC50 is much higher compared to concentrations in the 2D results.

Proliferation and apoptosis was assessed performing IHC for Ki-67 and cleaved caspase-3, respectively. The proliferation decreased after treatment whereas apoptosis was increased, showing a prominent temperature and duration dependent effect (**Figures 4E-H**).

Overall, all these *in vitro* results show a temperature- and duration- dependent efficacy for both oxaliplatin and MMC.

### 3.2 A stable body weight and stable temperature distributions were achieved using the semi-open four-inflow HIPEC setup

After tumor outgrowth throughout the peritoneum was confirmed using ultrasound, the HIPEC treatment was applied. On the day of treatment, the overall average body weight was 148.6 gram with a range of 119.4-192.7 gram. The relative body weight was slightly decreased, but stable after treatment and between the different treatment groups the body weight was not significantly different (**Figures 2G, H**).

The inflow temperature for the 38°C group was 37.6°C (SD=0.26) and for the 42°C group 41.7°C (SD=0.37). Only small temperature differences were observed between treatment groups with the same inflow temperature but with different durations (**Supplementary Figure 1**). The average achieved temperatures in the peritoneum ranged from 36.95-37.63°C and 40.51-41.37°C in the 38°C and 42°C group, respectively.

Overall the rectal temperature was higher than in the esophagus, which was expected since the rectum is close to the heated region, but not exceeded the limit temperature of 38°C (**Supplementary Figure 1**).

During the follow-up of two days, the average relative body weight was decreased in all treatment groups both 24 and 48 hours after HIPEC, but less than 10% weight loss (**Figures 2G, H**). The largest decrease in body weight for the oxaliplatin-based HIPECs was measured in the 90 min 42°C treatment group 48 hours after treatment (95.53%; with a range of 90.18-100.95%). For the MMC-based HIPEC the smallest difference in body weight was observed in the 60 min 42°C treatment group (94.04%; with a range of 90.78-96.76%).

By using the semi-open four-inflow HIPEC setup in rats, a stable temperatures distribution can be achieved, with minimal signs of discomfort during follow-up, even using elevated temperatures.

### 3.3 Extent of disease was not affected shortly after HIPEC treatment

The average overall extent of disease was similar in both the oxaliplatin and MMC treatment groups, 13 in a range of 8-22 and 13.4 in a range of 7-21, respectively. PCI scores between different treatment groups using the same drug did not show any significant difference (**Figures 3C, D**).

Regions 2, 0, 4, 6 and 8 were the most common sites for tumor lesions formation (**Figure 3B**). The vast majority of the tumor lesions were observed in the cranial part of the abdomen, mainly at the liver hilum, lesser gastric curvature, perisplenic, and on the omentum. In **Figure 3E** pictures of the abdomen are presented with the corresponding PCI score. In the upper panel, specifically the tumor lesions on the omentum (region 0) are shown, where often a large number of very small (1–4 mm) tumor lesions were found. Tumor lesions at other sites than the omentum are presented in the lower panel. Tumor lesions in region 2, at the liver hilum and gastric curvature, were measured in a size ranging from 3-6 mm. Tumor lesions with a slightly larger size (4–8 mm) occurred in region 4, 6 and 8, in the intestine region and close to the kidneys. Overall, the extent of disease was not affected when measured shortly after HIPEC treatment.

### 3.4 Increased oxaliplatin uptake after a 90-minute HIPEC at 42°C

**Figure 5:**
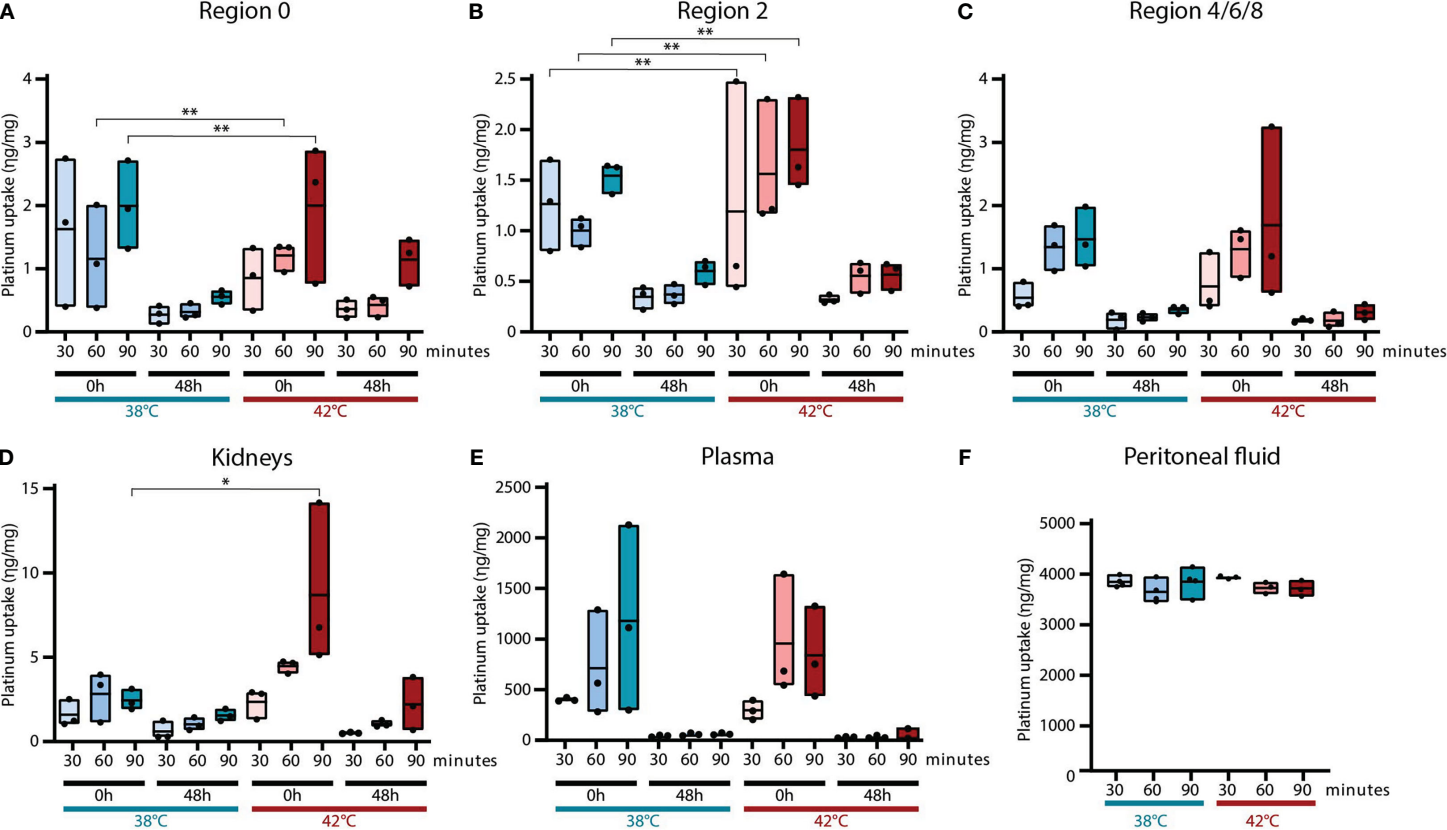
Platinum uptake in tumors and normal tissue. The platinum uptake was measured using ICP-MS in tumor lesions, kidneys and plasma collected 0 hours and 48 hours after HIPEC treatment. Tumor samples were collected from three different regions: 0 **(A)**, 2 **(B)** and 4/6/8 **(C)**. To define the normal tissue toxicity, platinum levels were also measured in the kidneys **(D)** and plasma **(E)**. Platinum levels in peritoneal fluid collected at the end of each HIPEC procedure was also analyzed **(F)**. Values of 3 samples are presented with the min/max ± mean. Significant p-values are reported as * <0.05 and **<0.01.

Overall, higher platinum levels were observed in samples collected directly (0h) after HIPEC, compared 48 hours after treatment (**Figures 5A-C**). The highest uptake was measured in the tumor lesions of rats treated for 90 minutes with an inflow temperature of 42°C. In this group, the platinum levels at 0h were 2.0, 1.7 and 1.7 ηg/mg, and decreased to 1.2, 0.5 and 0.4 ηg/mg when collected 48h after treatment, in region 0, 2 and 4/6/8, respectively.

Pilot measurements showed only an increased uptake of platinum in the kidneys and not in the liver, stomach, spleen and bowel. In rats treated for 90 minutes in combination with an elevated inflow temperature, a significantly higher platinum uptake was measured in kidneys collected 0h after treatment (**Figure 5D**).

The high platinum levels in the plasma samples taken directly after treatment are decreased to the minimum 48 hours after HIPEC treatment (**Figure 5E**). Mainly a longer duration resulted in more platinum uptake.

Equal platinum levels were observed in peritoneal fluid collected at the end of different HIPEC procedures (**Figure 5F**). This confirms that the concentration of oxaliplatin did not differ throughout the different oxaliplatin treatment groups.

An increased inflow temperature and a long duration during oxaliplatin-based HIPEC, result in a higher platinum concentrations in tumor nodules present in the peritoneum, but also in the kidneys and blood.

### 3.5 Elevated temperatures and longer treatment durations during oxaliplatin-based HIPECs result in more apoptosis and less proliferation

**Figure 6:**
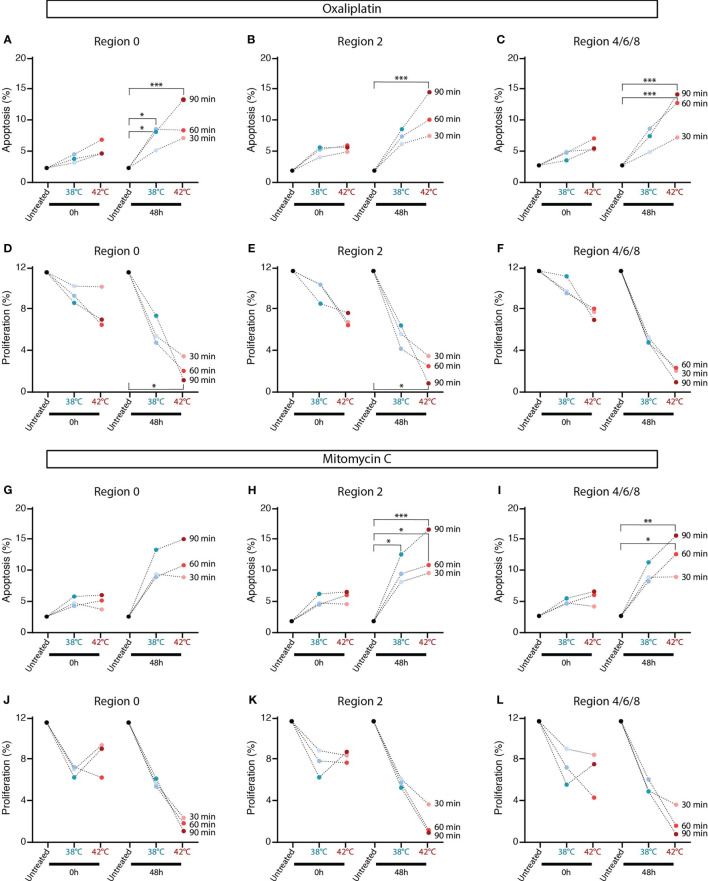
In oxaliplatin and mitomycin-C (MMC) HIPEC treated rats increased tumor cell apoptosis and reduced proliferation was observed. Percentages apoptosis in tumor lesions collected 0 hours or 48 hours after oxaliplatin- **(A-C)** and MMC-based **(G-I)** HIPEC treatment were analyzed using immunohistochemistry staining for cleaved caspase-3. Percentages proliferation in tumor lesions collected 0 hours or 48 hours after oxaliplatin- **(D-F)** and MMC-based **(J-L)** HIPEC treatment were analyzed using immunohistochemistry staining for Ki-67. Means of 4-5 animals are presented. The dotted line connect means with the same treatment duration. Significant p-values are reported as * <0.05, **<0.01 and ***<0.001.

Apoptotic levels are slightly increased directly after oxaliplatin-based HIPEC treatment, but 48 hours after treatment, an increase in apoptosis levels were observed (**Figures 6A-C**). Apoptotic levels were increased when oxaliplatin was applied with an inflow temperate of 42°C instead of 38°C in all tumor samples two days after treatment (38°C *vs*. 42°C/90-min; 7.14% *vs* 12.6%). Significantly more apoptosis was observed in all regions after oxaliplatin was applied at 42°C for 90 minutes compared to untreated tumors. Also a longer duration affects the treatment result as reflected by overall more apoptosis, in particular in samples collected 48 hours after treatment when treated at 42°C (30-min *vs*. 90-min/42°C; 6.44% *vs*. 16.6%).

Oxaliplatin-based HIPECs at 42°C resulted directly in less proliferation, but proliferation was mainly decreased in tumors collected 48 hours after treatment (**Figures 6D-F**). Significantly less proliferation was observed after oxaliplatin was applied at 42°C for 90 minutes compared to untreated tumors. Overall, the lowest proliferation levels after a 90-minute HIPEC at 42°C (38°C *vs*. 42°C/90-min; 6.73% *vs*. 1.9%). Overall, a longer duration resulted in less proliferation, but particularly when treated at 42°C (30-min *vs*. 90-min/42°C; 6.7% *vs*. 1.9%).

An overview of representative IHC stainings for cleaved caspase-3 and Ki-67 on tumor lesions are presented in **Supplementary Figure 2** for treated and **Supplementary Figure 3** for untreated samples. Both a higher treatment temperature and a longer treatment duration positively affects the HIPEC treatment outcome with more apoptosis and less proliferation in tumor lesions present throughout the peritoneum of rats.

### 3.6 MMC-based HIPEC is more effective when applied at higher temperatures and longer durations

Similar results were obtained in tumor samples collected after MMC-based HIPEC treatments. Directly after treatment, the apoptotic levels were minimally increased, while samples collected 48 hours later show increased apoptosis, especially when treated at 42°C for 90 minutes (**Figures 6G-I**). Elevated temperatures increase the apoptosis levels 48 hours after treatment (38°C *vs*. 42°C/90-min; 11.23% *vs*. 15.3%). A longer treatment duration resulted in elevated apoptotic levels, in particular in samples collected 48 hours after treatment and treated at 42°C (30-min *vs*. 90-min/42°C; 8.19% *vs*. 14.32%).

MMC-based HIPECs resulted directly in lower proliferation levels, but more effect was observed in the samples collected 48 hours after treatment (**Figures 6J-L**). The proliferation levels decreased more when HIPEC was applied at 42°C (38°C *vs*. 42°C/90-min; 6.1% *vs*. 2.0%). Mainly in the 48 hours collected samples treated at 42°C, a longer duration resulted in less proliferation (30-min *vs*. 90-min/42°C; 4.02% *vs*. 1.9%).

Overall, more apoptosis and less proliferation was observed in tumor lesions collected from rats receiving HIPEC at a higher treatment temperature and longer treatment duration.

### 3.7 Normal tissue toxicity

Normal tissue toxicity in rats after HIPEC was assessed in the liver, stomach, spleen, kidneys and bowel. Organs were collected two days after treatment and stained for apoptosis and proliferation. Representative pictures of the liver, stomach, spleen, kidneys and bowel stained for cleaved capase-3 and Ki-67 after HIPEC treatment are presented in **Supplementary Figure 4**. Representative pictures of untreated tissue is presented in **Supplementary Figure 3**. Overall, the number of cells in apoptosis and proliferation were similar throughout the different treatment regimens for both oxaliplatin- and MMC-based HIPECs and compared to untreated samples.

## 4 Discussion

The role of HIPEC for PMCRC is currently under debate since no survival benefit was shown in the PRODIGE-7 trial that randomized between CRS alone and CRS with the short oxaliplatin-based HIPEC schedule in PMCRC patients who were pre-treated with systemic chemotherapy (19). The HIPEC procedure was applied using high-dose oxaliplatin with a short duration of 30 minutes at a temperature of 43°C in an open or closed delivery technique. In the CRS with HIPEC group, no survival benefit and increased morbidity was shown compared to CRS alone. An issue with the PRODIGE-7 protocol is that patients also received systemic oxaliplatin prior to CRS, possibly rendering them less sensitive to oxaliplatin during HIPEC. This HIPEC schedule was also used in a prophylactic setting in the randomized COLOPEC and PROPHYLOCHIP trials, which were both negative as well (17, 18). Some argue that the results of the COLOPEC trial should not be analyzed using Intention to Treat analysis, since only 87 out of 100 patients received the adjuvant HIPEC treatment in the experimental arm with 9 patients ineligible as they were found to have an established metastasis at the start of the intended HIPEC procedure (38). Performing a Per Protocol analysis comparing the 87 patients receiving adjuvant HIPEC to the 102 patients receiving no HIPEC in the control arm does show a significant protective role of HIPEC against PM recurrence. Some conclude from these results that HIPEC does not improve the outcome of CRS for PMCRC patients (39). This conclusion may be premature, as the negative treatment outcome in the HIPEC group can be due to a suboptimal choice of chemotherapy, carrier solution, treatment duration and temperature. It can be argued that the short duration of a 30-minute HIPEC treatment was insufficient, since the treatment duration in most positive trials is 90 minutes (40). HIPEC duration in the protocols of past and ongoing MMC-based HIPEC trials for PMCRC patients is 60 or 90 minutes (9, 41).

In *in vitro* experiments the temperature- and duration- dependent efficacy for both oxaliplatin and MMC indicated that both a higher temperature and a longer treatment duration positively influence the efficacy of both chemotherapeutic agents in the CMS4-like CC-531 CRC cells and organoids. These data were confirmed in a representative preclinical *in vivo* model to make the translation step to the clinic. Our in house developed semi-open four-inflow HIPEC setup resulted in a stable and equal temperature distribution throughout the peritoneum. Overall, rats showed no signs of discomfort after treatment and the endpoint after HIPEC was easily reached. Only a small decrease in minimal body weight (<10%) was observed, and normal tissue toxicity was not seen.

A small amount of rats (6%) were sacrificed before reaching the endpoint because of signs of discomfort. Most of those animals were included in the first treatment batches of this study, in which the researchers were still gaining experience in performing the HIPEC procedure. From this, we concluded that gaining experience on the application of HIPEC in rats was important, in particular to carry out the procedure efficiently and to learn to control the body temperatures. After this learning curve it is feasible to apply HIPEC in rats with a stable temperature throughout the whole peritoneum, without discomfort for the animals during follow-up, even when using elevated temperatures during HIPEC.

After performing the HIPEC treatment in the rats, apoptosis and proliferation levels were assessed in tumor lesions, healthy tissue and blood. Mainly tumor lesions collected 48 hours after treatment showed that elevated temperatures and a longer treatment duration resulted in increased apoptosis levels and decreased proliferation rate. As expected, the collected samples immediately after treatment showed minimal effect. Tumors were collected from different peritoneal regions, but these did not show differences, confirming a stable and homogeneous drug and temperature distribution during HIPEC.

To achieve optimal treatment conditions, it is important to achieve a stable temperature and drug distribution to ensure an homogenous heat and drug exposure throughout the entire peritoneal cavity. The number and position of the in- and outflow catheters play an important role in the application of HIPEC. Numerical modelling based on computational fluid dynamic software showed that increasing the distance between the in- and outflow, using more inflows and increasing the flow rate resulted in better peritoneal fluid distributions (42, 43). These findings were confirmed by Loke et al. using four inflow catheters instead of one to perform HIPEC in rats, resulting in a more stable and more homogeneous thermal distributions (35).

The drug uptake was only assessed on oxaliplatin treated samples. Also in these samples, the application of a higher temperature and a longer duration during HIPEC positively affected the results. Higher platinum levels were not only measured in the tumor nodules, but also in the kidneys and blood. These levels were decreased within the follow-up of two days. Unfortunately, it was not possible to measure the uptake of MMC since there is no validated protocol. The uptake of platinum-based drugs is relatively easy to measure, because the active platinum component of these pharmaceuticals is not influenced by the tissue digestion protocol, which is necessary to carry out a quantitative ICP-MS determination. The active component of MMC is less evident to quantitatively extract from the tissue samples, which makes the liquid chromatography-mass spectrometry (LC-MS) based measurements and uptake data less reliable.

Although current results focus on short-term effects, our study does contribute new insights towards improving clinical application of HIPEC. The treatment parameters treatment duration and temperature of the perfusate have a strong effect on the effectiveness of both oxaliplatin- and MMC-based HIPEC treatments in a relevant rat model. Our data suggest that a treatment duration of 60 or 90 minutes with a high inflow temperature (42°C) is crucial for a successful treatment outcome for both MMC- and oxaliplatin-based HIPEC treatment protocols. Some clinical trials applying oxaliplatin-based HIPEC in PMCRC patients showed no prolonged survival using a 30 minutes treatment duration at a temperature of 43°C (17–19). A longer treatment duration of 60 and 90 minutes, resulted in our rats in more platinum uptake, less proliferation and higher levels of apoptosis, showing a strong duration-dependent efficacy. It is important to consider a 60 or even a 90-minute treatment duration for oxaliplatin-based HIPEC treatment protocols, instead of 30 minutes, in PMCRC patients to achieve a prolonged survival.

Hyperthermia has an effect on both the tumor and microenvironment (44) with biological effects such as an increased blood flow, inhibiting the DNA repair pathways, increase of heat-shock proteins, cell membrane permeability and activating the immune system resulting in more drug uptake (45–48). The effectiveness of hyperthermia has strong dose-effect relationship with temperature and duration. Prolonging a 30-minute chemotherapy exposure at 42°C to 60 minutes, improves the treatment effectiveness of oxaliplatin in a preclinical micrometastasis model and the cisplatin growth-inhibitory effect in gastric cancer cells, showing a temperature-dependent effectiveness (24, 49). *In vitro* studies show a strong temperature-dependent effectiveness, based on increased drug uptake, DNA damage and apoptosis at elevated temperatures (>41°C) alone or in combination with chemotherapeutic drugs (21, 50).

To follow up on the current study, it is important to assess also the long-term clinical effect of the different HIPEC treatment parameters. Although higher apoptosis and lower proliferation levels were quantified after HIPEC treatment in rats, the extent of disease was not affected within the relatively short follow-up after treatments. To evaluate the long-term effect on the tumor lesion outgrowth, it is necessary to extend the follow-up time after treatment with a couple of weeks, and to add CRS before HIPEC as well for removing too large tumor lesions. When assessing the long-term effect of elevated temperatures and longer treatment durations on the tumor outgrowth, it would also be relevant to explore the possible effect of HIPEC on the immune response. One of the effects of hyperthermia is the activation of an immune response by stimulating CD8+ effector T-lymphocytes, macrophages, dendritic cells, B-cells and natural killer cells (44, 51–54).

In conclusion, the efficacy of oxaliplatin and MMC during HIPEC in *in vitro* and *in vivo* PMCRC models is dependent on the temperature of the perfusate and the treatment duration. HIPEC applied with an elevated temperature of 41-42°C and a treatment duration of 90 minutes improves drug uptake, induces apoptosis and decreases proliferation in tumor of a PMCRC rat model in comparison to lower temperatures and shorter duration of exposure, without enhanced normal tissue toxicity.

## Data availability statement

The Illumina Sequencing data belonging to the manuscript are submitted and published now at: GSE226558 - GEO Accession viewer (https://www.ncbi.nlm.nih.gov/geo/query/acc.cgi?acc=GSE226558).

## Ethics statement

All experiments were approved by the Dutch Central Committee of Animal Experiments, with approval code AVD1180020174184, received on 14 February 2019, and carried out in accordance with the Dutch Animal Welfare Act 1997.

## Author contributions

Conceptualization: RH, NF, HK, PT, JC and AO. Formal analysis: RH, BB, AT, DL and AO. Investigation: RH, BB, GB, HR, ET, WM and AT. Data curation: RH and AO. Writing—original draft preparation: RH. Writing—review and editing: RH, BB, GB, HR, ET, WM, AT, DL, NF, HK, PT, JC and AO. Visualization: RH, JC and AO. Supervision: NF, HK, PT, JC and AO. Project administration: JC. Funding acquisition: NF, HK, JC, PT and AO. All authors contributed to the article and approved the submitted version.
