# Supplementary figures and images for: Elevated temperatures and longer durations improve the efficacy of oxaliplatin- and mitomycin C-based hyperthermic intraperitoneal chemotherapy in a confirmed rat model for peritoneal metastasis of colorectal cancer origin
Source: Front Oncol. 2023 Mar 16;13:1122755. doi: 10.3389/fonc.2023.1122755 (PMC10064448; doi:10.3389/fonc.2023.1122755)

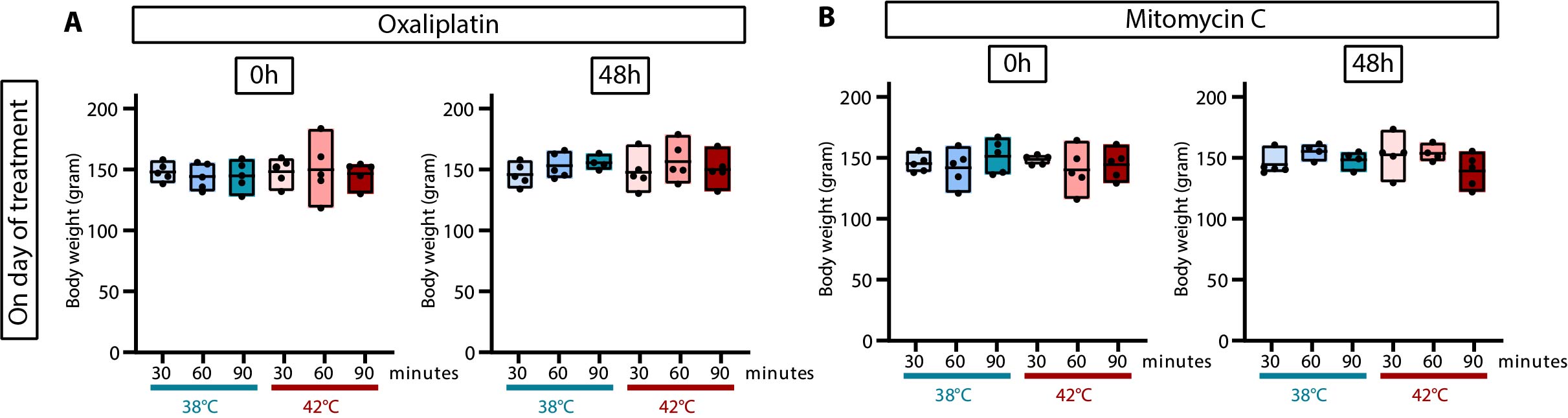

Supplement: Supplementary file 1 [file Image_1.jpeg]

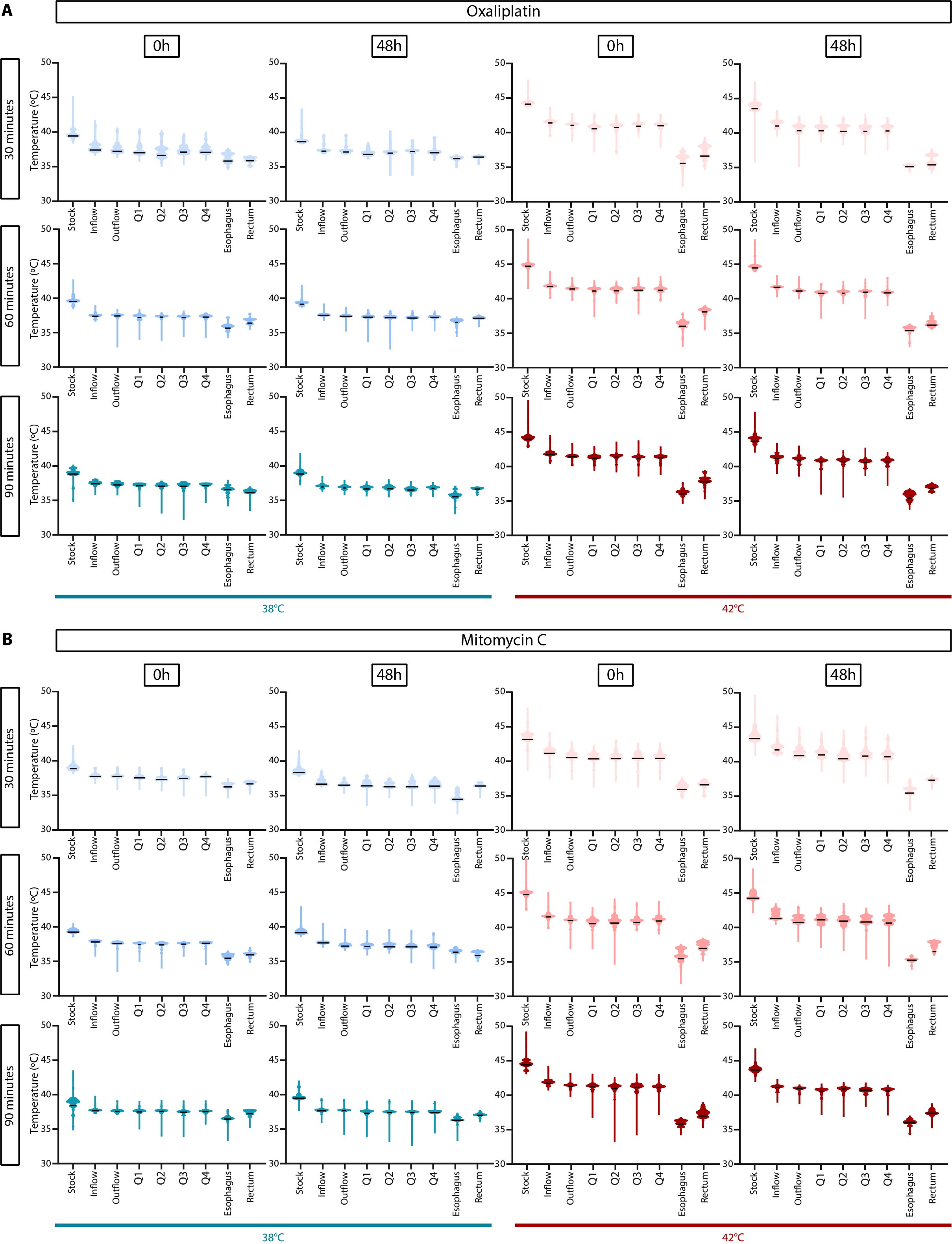

Supplement: Supplementary file 2 [file Image_2.jpeg]

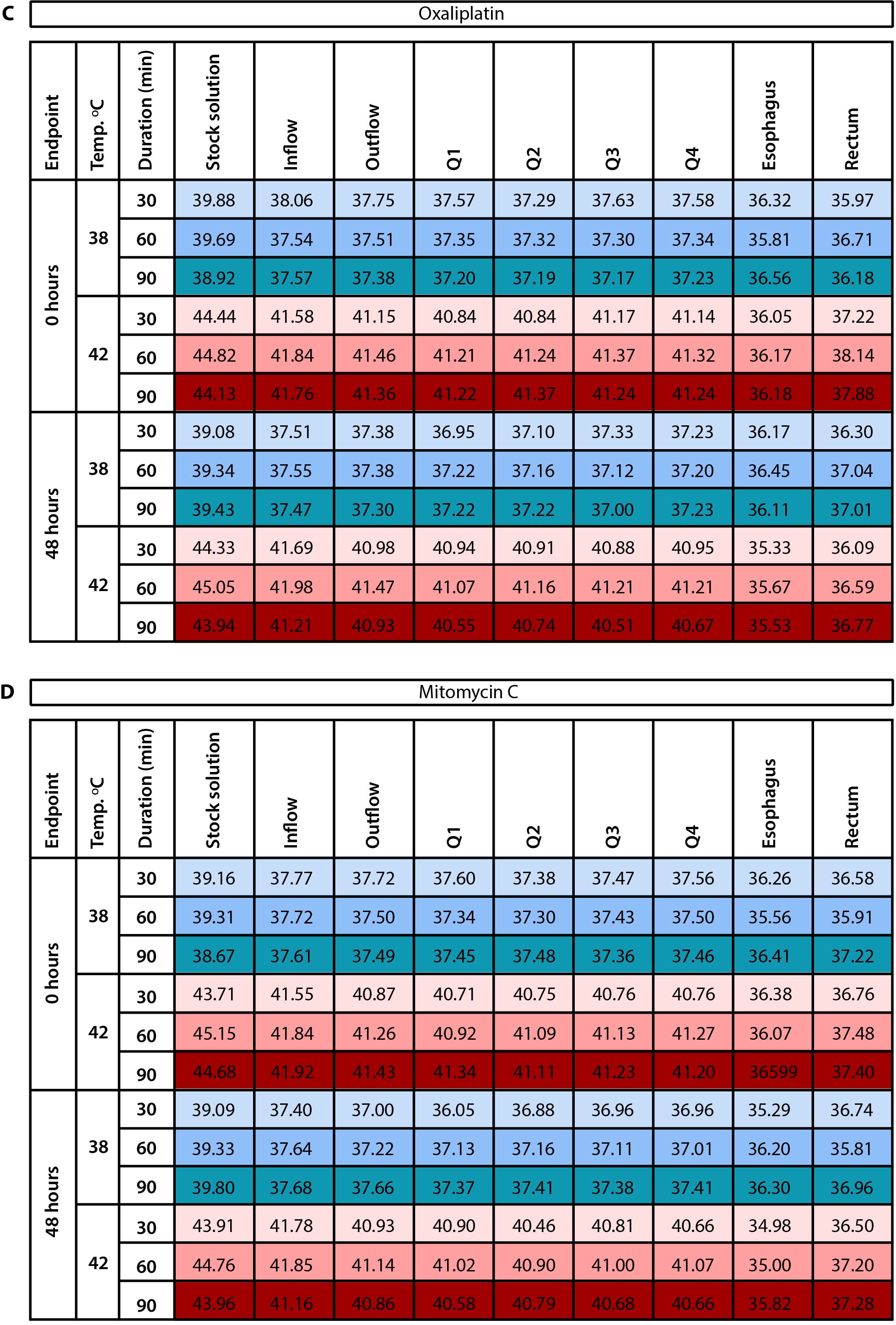

Supplement: Supplementary file 3 [file Image_3.jpeg]

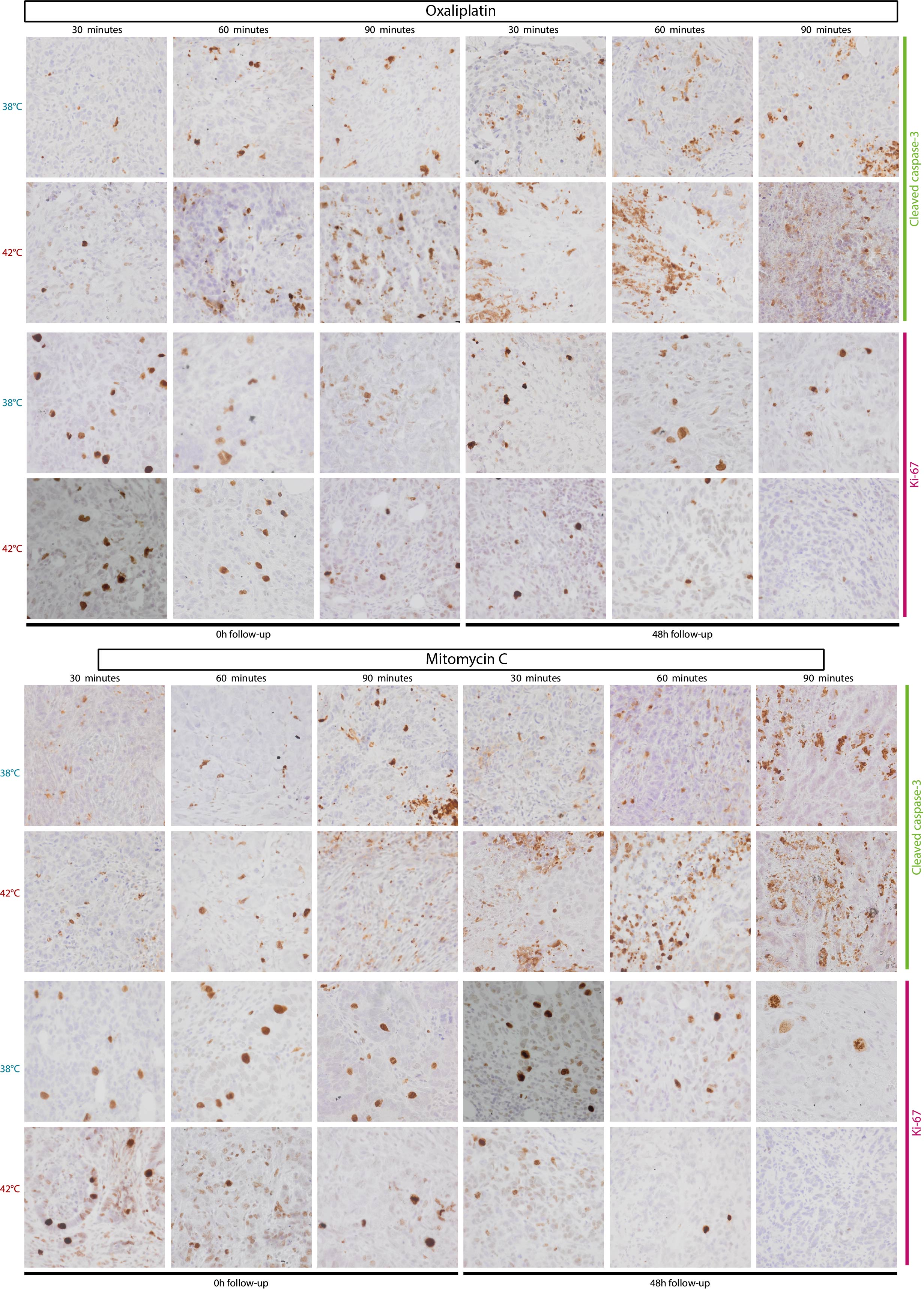

Supplement: Supplementary file 4 [file Image_4.jpeg]

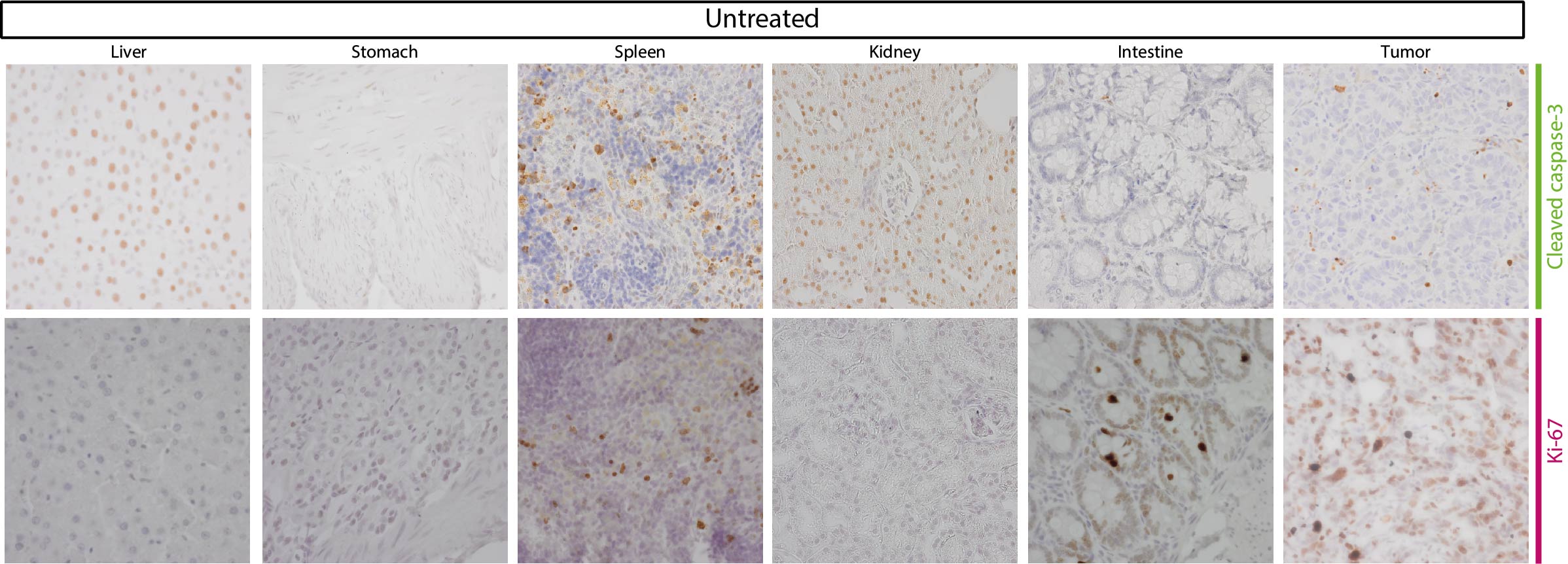

Supplement: Supplementary file 5 [file Image_5.jpeg]

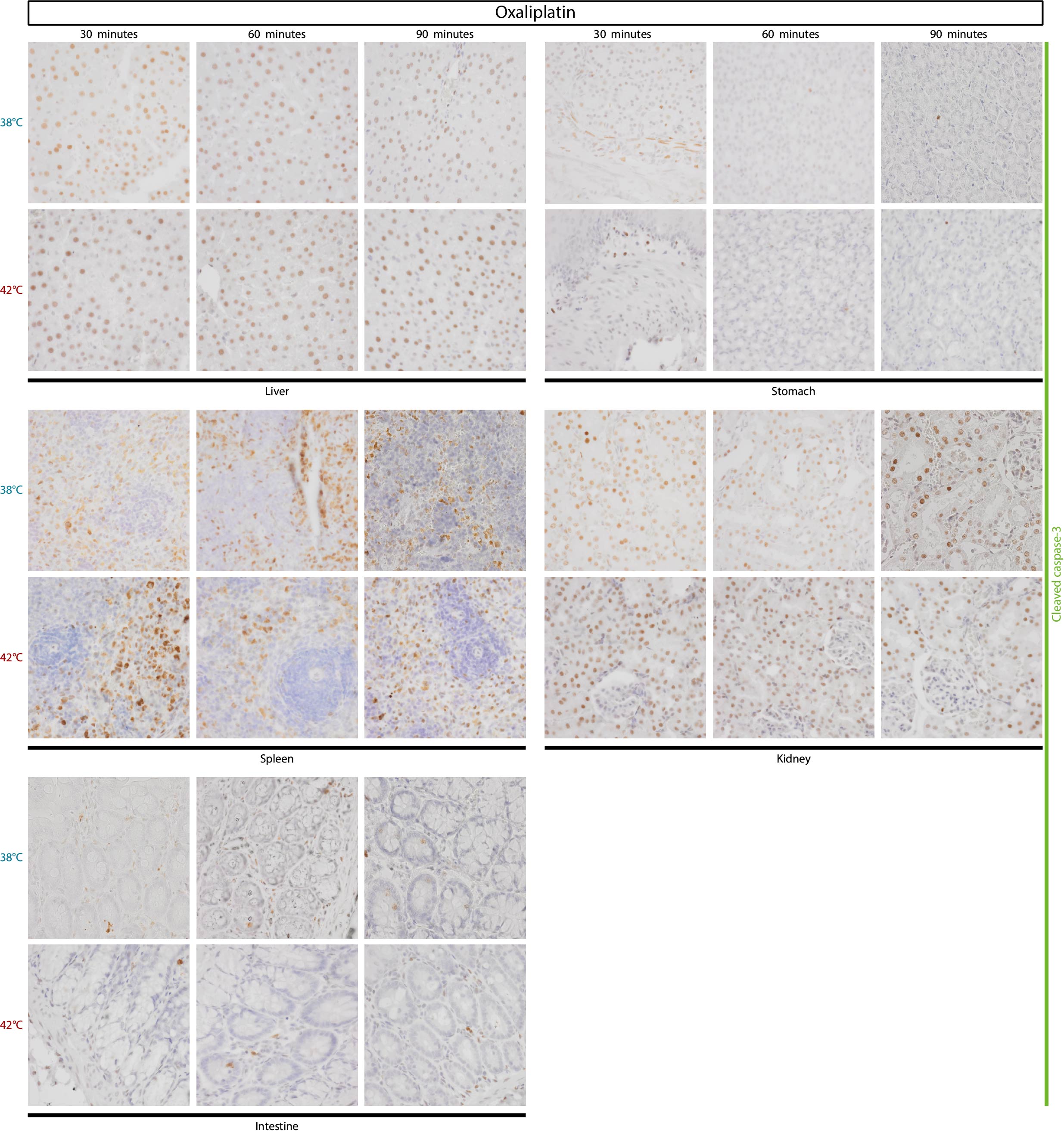

Supplement: Supplementary file 6 [file Image_6.jpeg]

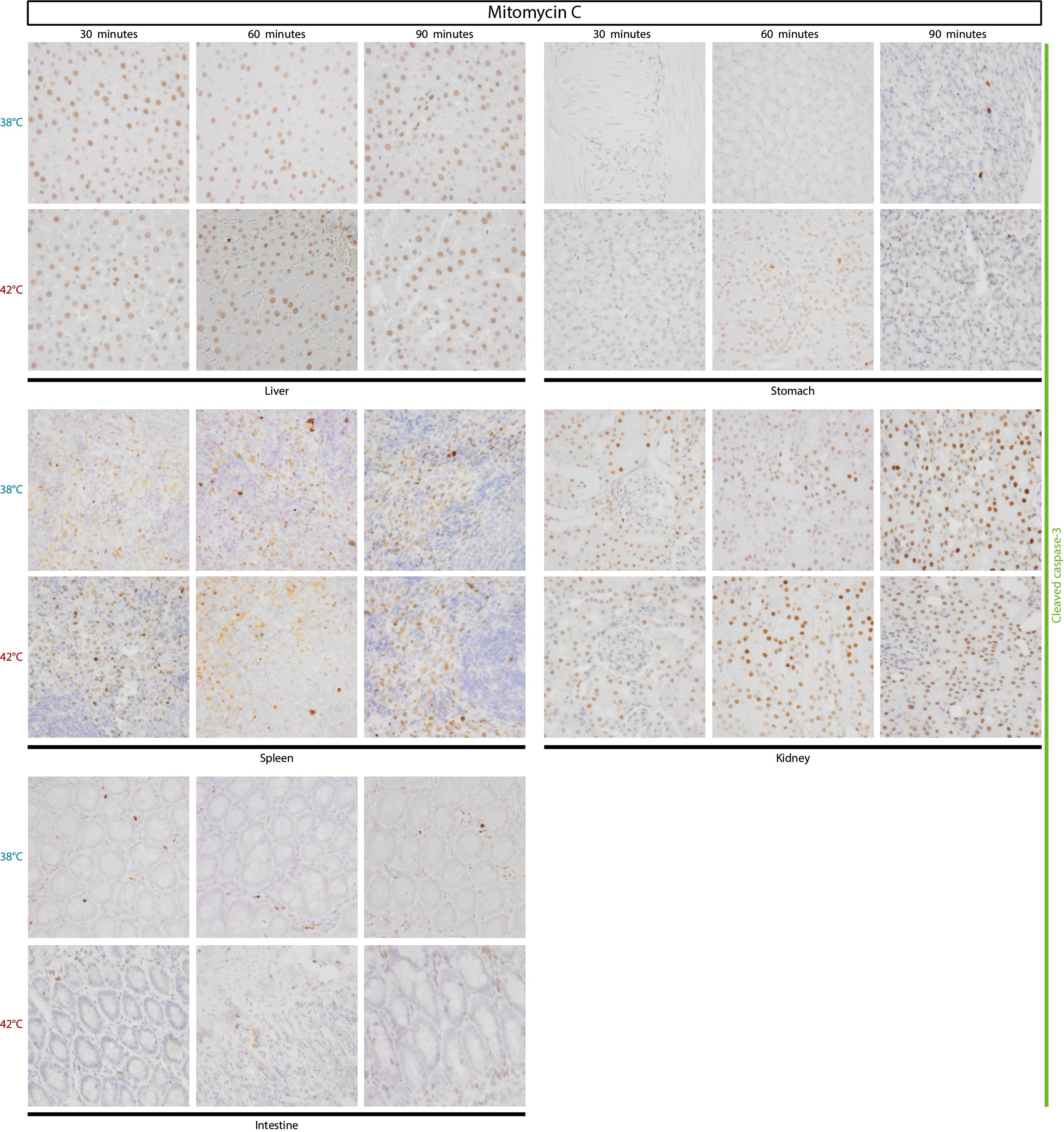

Supplement: Supplementary file 7 [file Image_7.jpeg]

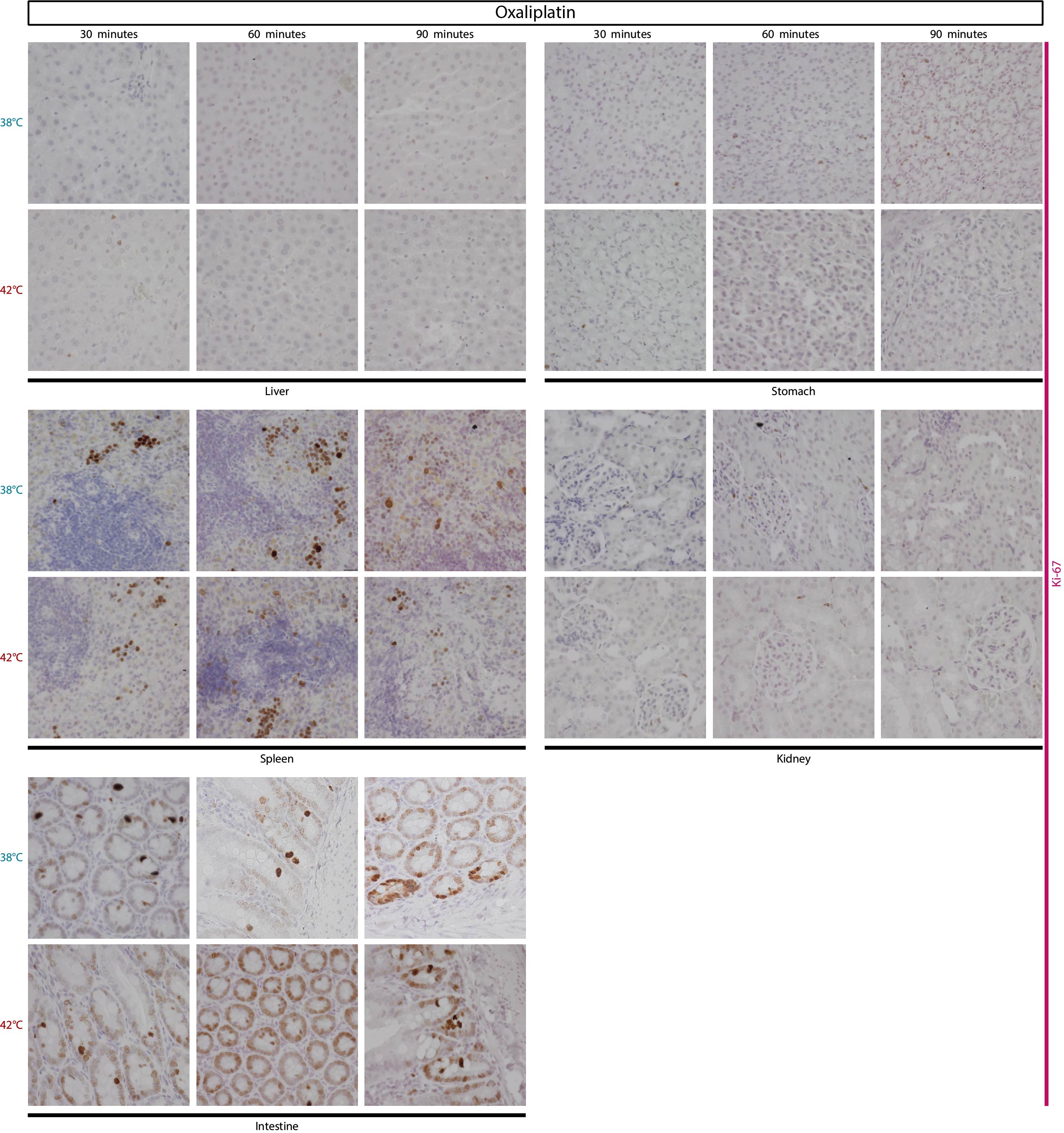

Supplement: Supplementary file 8 [file Image_8.jpeg]

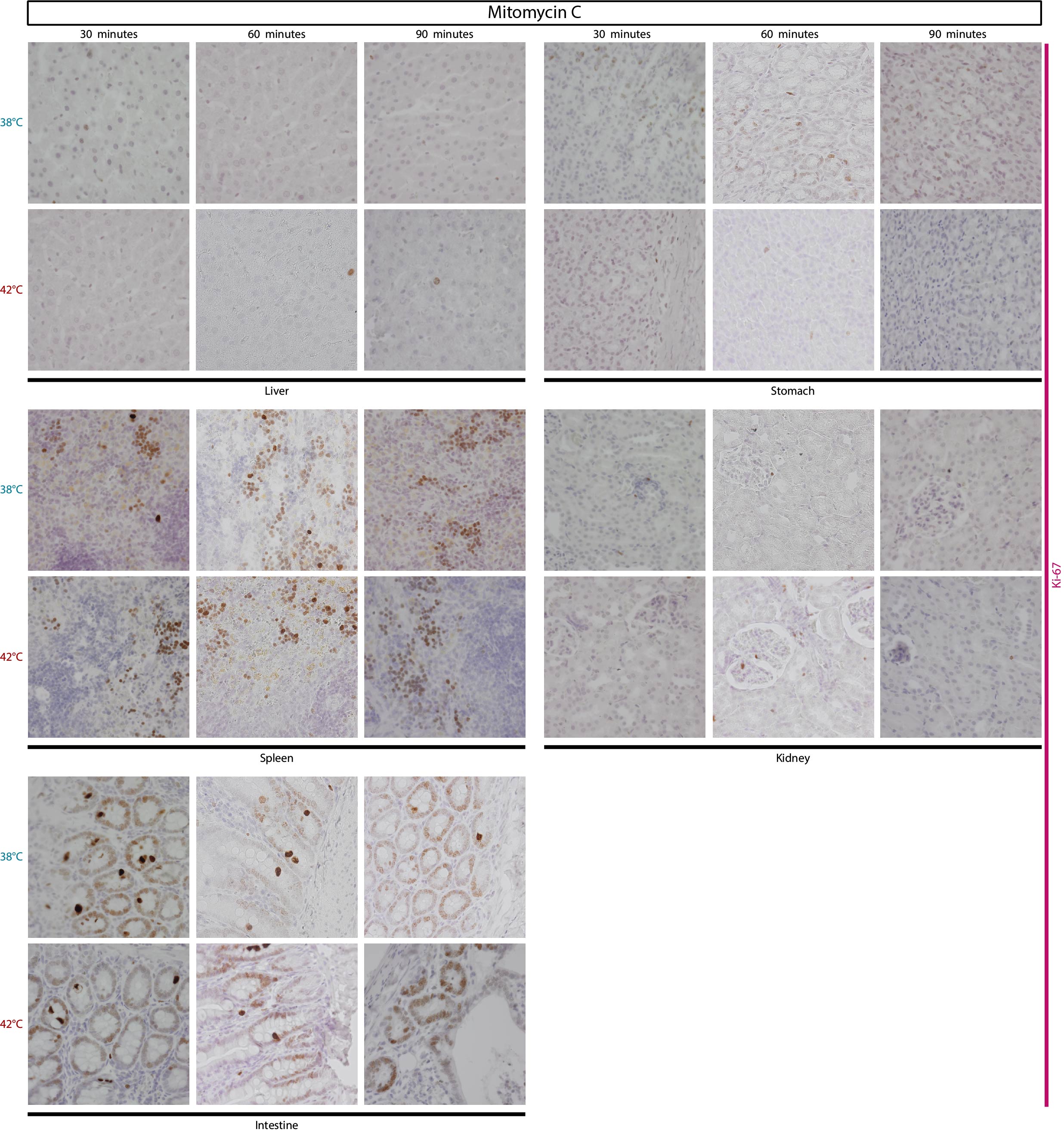

Supplement: Supplementary file 9 [file Image_9.jpeg]
